# NG-GAN: A Robust Noise-Generation Generative Adversarial Network for Generating Old-Image Noise

**DOI:** 10.3390/s23010251

**Published:** 2022-12-26

**Authors:** Sadat Hossain, Bumshik Lee

**Affiliations:** Department of Information and Communication Engineering, Chosun University, Gwangju 61452, Republic of Korea

**Keywords:** generative adversarial network, image denoising, recurrent residual channel and spatial attention, noise generation, perception-based image quality evaluator

## Abstract

Numerous old images and videos were captured and stored under unfavorable conditions. Hence, old images and videos have uncertain and different noise patterns compared with those of modern ones. Denoising old images is an effective technique for reconstructing a clean image containing crucial information. However, obtaining noisy-clean image pairs for denoising old images is difficult and challenging for supervised learning. Preparing such a pair is expensive and burdensome, as existing denoising approaches require a considerable number of noisy-clean image pairs. To address this issue, we propose a robust noise-generation generative adversarial network (NG-GAN) that utilizes unpaired datasets to replicate the noise distribution of degraded old images inspired by the CycleGAN model. In our proposed method, the perception-based image quality evaluator metric is used to control noise generation effectively. An unpaired dataset is generated by selecting clean images with features that match the old images to train the proposed model. Experimental results demonstrate that the dataset generated by our proposed NG-GAN can better train state-of-the-art denoising models by effectively denoising old videos. The denoising models exhibit significantly improved peak signal-to-noise ratios and structural similarity index measures of 0.37 dB and 0.06 on average, respectively, on the dataset generated by our proposed NG-GAN.

## 1. Introduction

Image denoising primarily aims to eliminate unwanted signals from noisy observations. Considerable research has been conducted in this field, which is considered one of the most fundamental vision issues [1,2,3]. Significant advances have been made in image denoising with the advent of deep learning. Although deep convolutional neural networks (CNNs) for image enhancement have shown promising results [4,5,6,7,8,9,10,11,12,13,14,15], several crucial obstacles prohibit their deployment in real-world applications. Because learning-based techniques are typically data-driven, training on a given dataset does not always ensure generalization to real-world scenarios. For various reasons, noise from a typical camera pipeline differs from the theoretical noise assumption. For example, common additive white Gaussian noise (AWGN) implies that the term is signal-independent [16,17], which differs from actual noise. Hence, when a denoising algorithm is trained on synthetic data, such as AWGN, generalizing it to image restoration is difficult. Executing learning-based algorithms on a significant number of high-quality datasets is crucial. Most conventional learning-based denoising methods focus on the traditional Gaussian denoising problem and pay more attention to the architecture design of deep learning networks because creating a pair of noisy and noise-free images is simple using additive synthetic noise. In [18,19], well-aligned noisy and clean image pairs with real-world noise were collected, allowing denoising algorithms to be learned in a supervised manner. Although such a technique successfully addresses real-world noise, obtaining large-scale pairings remains challenging due to two main practical difficulties. First, this is because of the lack of denoised or enhanced versions of old images. In addition, old images are more likely to degrade in a more complicated manner than modern images. Second, no degradation model can accurately depict the artifacts of old images because the network cannot approximate them because of the domain disparity between synthetic and actual old images.

Generation-based techniques have been developed to address these issues [20,21]. These methods employ noisy target images to train a noise generator, producing pseudo-noisy images coupled with clean images that are then used to train a denoising model. Following the success of earlier synthetic noise reduction technologies, attempts have recently been made to adapt this technology to real-world noise [22]. However, no generation-based solution that properly imitates real-world noise has been proposed without supplying associated clean pictures to the target noisy images. 

Gaussian and digital camera noise are insufficient for creating noise for the old film; generating global noise artifacts that can alter the contrast and brightness of the entire frame must be possible, as well as local noise that affects only a small area of the image. Actual old images are significantly more difficult to generate accurately because they frequently suffer severe deterioration from various unknown degradations. Furthermore, with technological advancements, current digital cameras are considerably more advanced in capturing the subtle characteristics of images than old cameras. Thus, images captured with modern cameras are unlikely to contain similar noise, distortion, or artifacts to those of old images. Hence, the collection of datasets for paired old and clean images is a challenging task. 

This paper proposes a noise-generation generative adversarial network (NG-GAN), a noise-generation framework that can be trained without paired datasets. Using the perception-based image quality evaluator (PIQE) [23] metric with a clean image, noisy images can be generated in a more elaborately controlled manner. The following is a summary of the contributions of this study:

- We propose a noise-generation framework for old images and videos using a no-reference PIQE metric and an unpaired clean image to generate a noisy image based on the value of the PIQE metric.
- We introduce a recurrent residual convolutional and attention mechanism-based robust framework, NG-GAN, that successfully imitates the noisy pattern of degraded images.
- When state-of-the-art (SOTA) video restorers are trained on the datasets generated by the NG-GAN, they can effectively produce clean videos from noisy ones in terms of the peak signal-to-noise (PSNR) and structural similarity index measure (SSIM).

This paper is organized as follows: Section 2 describes the related works. In Section 3, the proposed NG-GAN architecture for old image generation is explained in detail. The experimental results and analyses are shown in Section 4. The paper is concluded in Section 5. 

## 2. Related Works

A min-max game between the generator and discriminator is defined in a generative adversarial network (GAN) [24]. The generator aims to provide compelling samples that deceive the discriminator, thereby allowing the generated samples to be distinguished from the ground truth. The GAN is also used for visual enhancement and restoration, such as in super-resolution [25], image inpainting [26], and style transfer [27]. The first widely used GAN-based paired image-to-image translator is the Pix2Pix GAN [28], the first unpaired image-to-image translator CycleGAN [29], and DualGAN converts images from one domain to the other [30]. Although they are used to map images from one domain to another, they struggle to generate fine noisy images for a given set of clean images. Instead of employing a single model, generation-based approaches use a two-stage pipeline to solve the denoising problem [16,20,21,22]. First, an unsupervised noise generator is trained to replicate the distribution of the actual noisy samples, allowing any clean picture to be translated into pseudo-noisy data. The synthesized input and target pairs are then used to train a denoising model in a straightforward manner. Similar to other conventional methods, this GAN model aims to approximate the probability distribution of real-world noisy images by treating images as samples. This image-level GAN does not finely or accurately learn the actual noise distribution because it does not emphasize that each pixel of a real noisy image is a random variable or that the real noise is spatio-chromatically associated. The NTGAN approach utilizes noise maps created by a camera response function in the denoising network [31]. The GAN2GAN approach uses improved noisy-patch extraction to provide more realistic noisy samples to train the denoising model [16]. The DA-Net model generates noisy and clean images by learning the joint distribution of clean-noisy image pairs [32]. All studies mentioned have been conducted with digital camera-captured images, whereas we focused on generating noisy images that match the old image noise and degradation.

Image-to-image translation methods, such as Pix2Pix, CycleGAN, and DualGAN, are well-known unsupervised image translation methods. The basic working principle is that the models learn the translation using paired and unpaired images from different domains. When such models are utilized to generate realistic old image noise, they tend to focus on generating general translations, such as image color. However, they fail to generate detailed information, such as the noise and texture of old images, which significantly differ from that in the synthetic dataset. Consequently, images generated by these models lose significant noise information and variation in the noise pattern. To overcome these limitations, we carefully designed the generator architecture by providing additional information with clean images, added loss functions, and modified the discriminator architecture to focus on generating realistic-looking old images.

Recently, deep learning has adopted attention algorithms to improve feature extraction [33]. For example, ECA-Net [34] employs a local cross-channel connection method without downscaling or adaptive kernel selection for one-dimensional convolutional networks. Several dual-attention mechanisms have been used in addition to these single-channel mechanisms. Using channel and spatial attention mechanisms, a convolutional block attention module (CBAM) was introduced to enhance relevant information and eliminate redundant and irrelevant information [35]. To increase the weights of the effective features of old images in the channel and pixel space, we employed the CBAM in our proposed old noisy image generation network. The CBAM in NG-GAN helps the network learn and focus more on important information. That is, the CBAM enables the network to precisely record different features to focus on the most informative aspects while creating degraded images, which helps retain image features and edges while generating old images.

## 3. Proposed Method

### 3.1. Problems in Degraded Old Images

Investigating the statistical characteristics of complex real-world noise is worthwhile for developing realistic noise using deep learning networks. Noise in old images typically emanates from sources in low-performance cameras in the early stages, such as electronic sensors, in-camera amplifiers, photon noise, quantization, and compression artifacts. When all these components are combined, the pixel-wise distortion is blended with a baseline clean signal to produce a noisy image, as expressed in (1):

(1) $$I^{n}=I^{c}+y,$$

where $I^{n}$ is the noisy image, $I^{c}$ is the clean image, and y is the pixel-wise distortion. Noise component y is commonly assumed as AWGN in traditional deep-denoising approaches [4,5]. In [18], although the noise model can reasonably approximate the actual noise, many investigations have shown that actual scenarios are significantly more intricate [23,36,37]. Therefore, we used a learning-based strategy to imitate real-world noise rather than handmade approaches to solve the problem without employing paired data. To replicate the pattern of real-world noise, the proposed architecture fully exploits the ben-efits of unsupervised learning.

**Figure 1:**
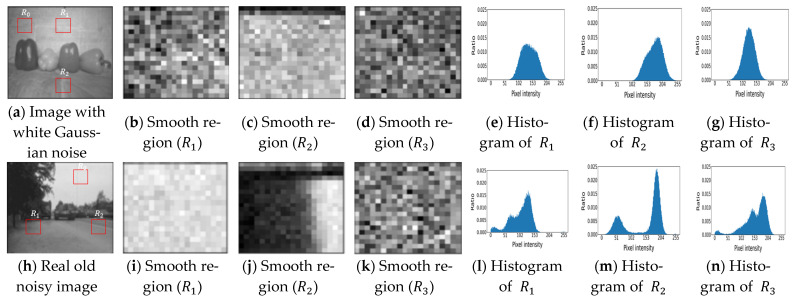
Histogram comparison between the smooth regions of AWGN added in a clean image and realistic old-image noise.

Figure 1 shows the histogram comparisons between AWGN and realistic old image noise. The smooth regions ($R_{1},R_{2},$ and $R_{3}$) from the AWGN-added images, and old noisy images are extracted, as shown in Figure 1a,h. The corresponding histograms show the difference between the distributions in Figure 1e–g,l–n. The histograms show that smooth regions with AWGN have a Gaussian-like distribution. In contrast, the histograms from the smooth regions of the old image noise show many small peaks with random distributions. The smooth region in Figure 1 is defined as pixel areas where the mean pixel value in the region approximates the pixel value itself. That is, R is a region in the image defined by $R\in R^{M\times N}$, and, if the intensity values of R are denoted by $I_{R}\left(x,y\right)$, we define a smooth region as any region satisfying $\overset{M}{\underset{x=1}{\sum }}\overset{N}{\underset{y=1}{\sum }}|E(I_{R})-I_{R}\left(x,y\right)|\;\approx \;0$. We assume that regions $R_{1},\;\;R_{2},$ and $R_{3}\;$ are smooth regions corrupted by a certain type of noise in the old images, and, in the AWGN-added image, they are corrupted by Gaussian noise. We approximate the noise in these regions using a histogram because these regions provide us with noise information.

### 3.2. Proposed Network Architecture

A denoising network attempts to recover the underlying clean signal from a given noisy observation if sufficient data pairs are used in supervised learning. However, for old image denoising, collecting clean-old noisy image pairs is challenging. First, clean images were collected from multiple sources, such as the REDS, PASCAL VOC, and DIV2K datasets [38,39,40]. Then, our proposed NG-GAN model is used to generate the target noise distribution, which can be obtained from the actual old images. In our proposed method, old images were also collected from the frames of old movies, such as D.O.A. (1949), Midnight Intruder (1938), A Matter of Life and Death (1946), and Bonjour Tristesse (1958). 

**Figure 2:**
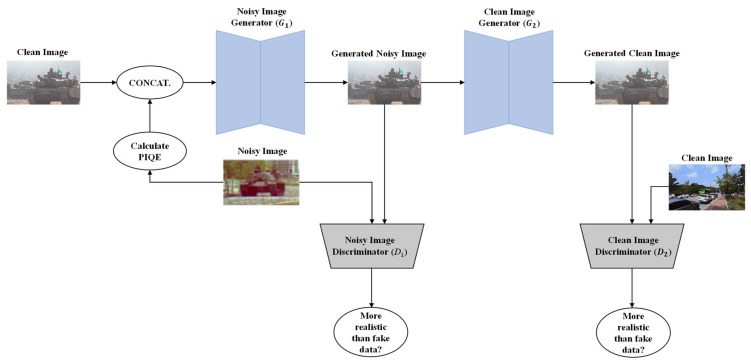
Overview of the proposed NG-GAN framework and its components, composed of two generators and two discriminators. The noisy image generator $G_{1}$ generates degraded images. The clean image generator $G_{2}$ reconstructs the clean version of the image. Noisy and clean image discriminators, $D_{1}$ and $D_{2}$, classify the fake and real images, respectively.

Figure 2 shows the overall framework of the proposed NG-GAN. The proposed NG-GAN was inspired by the CycleGAN framework [29]. CycleGAN has shown promising performance in color transformation and image transformation from one domain to another, such as sketch-to-photo photograph-to-Monet applications, as well as object transfigurations, such as in transfiguring a horse into zebra. In addition, CycleGAN helps obtain paired datasets using unpaired datasets. However, when CycleGAN was applied to generate old noisy images, our experimental investigation observed that the generated image showed a lack of variety in noise patterns and was likely to change the image geometry from the original image. It also struggled to separate an object from the context owing to its generator architecture and loss functions [41].

To address the problem of unpaired image-generating networks such as CycleGAN in generating realistic old noisy images, the PIQE metric was used as a no-reference PIQE to guide the network on the degradation quality of the old images in our proposed NG-GAN [23]. The VGG-19 and SSIM losses were used to guide the network in generating old noisy images well while maintaining the visual quality and structure of the images in the proposed NG-GAN [42,43]. A recurrent residual network strategy was used to better represent feature representation by accumulating features with the recurrent residual convolutional layers. In addition, the CBAM was adopted in the proposed NG-GAN to prevent the network from learning unnecessary background information. It also helps to learn and concentrate more on key information [35]. Moreover, the CBAM enables the network to accurately capture various features, pay attention to the most informative features, and then generate degraded images.

In summary, CycleGAN uses two cycles (A2B2A + B2A2B) to map images from one domain to another, whereas the proposed NG-GAN requires one cycle (A2B2A) for the same mapping, which saves a considerable amount of training time. Moreover, CycleGAN generates a similar type of noise pattern in the generated noisy images. To produce variety in the generated noise pattern, we concatenate random gaussian noise with the clean image to depict the stochastic behavior of noise in accordance with the condition of each scene. To overcome the problem of difficulty in retaining the structural information in CycleGAN, SSIM and VGG-19 losses are used in our proposed NG-GAN. The generator architecture of CycleGAN is inspired by Johnson et al. [44], and consists of 6 and 9 residual blocks used to generate images of size 128 × 128 and 256 × 256, respectively. The proposed method uses a U-Net shape architecture [45], where each block consists of two recurrent residual convolutional layer blocks (R2CL) that ensure better feature interpretation. We also integrated 1-D channel attention in each R2CL block to capture the correlation between channels. Finally, the proposed NG-GAN utilizes CBAM instead of skip connections and PIQE value extracted from old images, which help the network generate more realistic-looking old images.

As shown in Figure 2, the PIQE [23] values of the noisy images were obtained in the first step. The PIQE computes the no-reference quality score of an image using a block-wise distortion estimation. Initially, the mean subtracted contrast-normalized (MSCN) coefficient was calculated for every pixel in an image. The image was then divided into uniform-sized 16 × 16 blocks. Highly spatially active blocks were identified based on the variance of the MSCN coefficients. An activity mask was then obtained using the recognized spatially active blocks, representing the regions of the input image areas with higher levels of spatial variability caused by noise and compression artifacts. Subsequently, the MSCN coefficients were used to analyze the distortion caused by the blocking artifacts and noise in each block. A threshold criterion was used to classify distorted blocks with blocking artifacts, undistorted blocks, and blocks with Gaussian noise. Subsequently, the spatial quality mask of noticeable artifacts was generated from the distorted blocks with blocking artifacts, and the spatial quality mask of Gaussian noise was generated from the distorted blocks with Gaussian noise. Finally, the PIQE score of the input image was computed as the mean of the scores in the distorted blocks. 

The computed PIQE score of the noisy image was spatially replicated across all the pixel positions of $I^{C}$. Noisy image generator $G_{1}$ generates a noisy version of the clean image, depending on the PIQE value. The higher the PIQE value, the more noise it generates; the value ranges from 0 to 100. Clean image generator $G_{2}$ reconstructs the clean image from the fake noisy image generated by $G_{1}$. Two discriminators, $D_{1}$ and $D_{2}$, provide an approximation of how real or fake the generated noisy and clean images are, respectively. The losses used to train the NG-GAN can be expressed as in (2)–(4).

(2) $$l_{1}=\left|I^{c}-G_{2}\left(I^{g}\right)\right|$$

(3) $$l_{VGG/i.j}^{Rec}=\frac{1}{W_{i,j}H_{i,j}}\overset{W_{i,j}}{\underset{x=1}{\sum }}\overset{H_{i,j}}{\underset{y=1}{\sum }}{\left(\phi_{i,j}{\left(I^{c}\right)}_{x,y}-\phi_{i,j}{\left(G_{2}\left(I^{g}\right)\right)}_{x,y}\right)}^{2}$$

(4) $$l_{SSIM\;}=1-SSIM\;\left(I^{c},\;G_{2}\left(I^{g}\right)\right)\;\;$$

The generated noisy images should be as close as possible to the clean input images in terms of their structure. Hence, we adopt the $l_{1}$, $l_{VGG/i.j}^{Rec}$ and $l_{lSSIM\;}$ loss, where $l_{1}$ is the content loss measuring the $l_{1}$ norm distance between the reconstructed image $G_{2}\left(I^{g}\right)\;$ and original clean image $I^{c}$. The $l_{VGG/i.j}^{Rec}$ loss is based on the pre-trained 19-layer VGG network rectified linear unit (ReLU) activation layers. Indices i and j indicate the $i^{th}$ max-pooling layer and $j^{th}$ convolution (after activation) within the VGG-19 network, respectively. $\phi_{i,j}$ denotes the feature maps acquired by the $j^{th}$ convolution layer. $W_{i,j}$ and $H_{i,j}$ are the dimensions of respective feature maps in the VGG network. The Euclidean distance between the features extracted from the reconstructed and reference image is then defined as the VGG loss. The mean squared error treats every pixel as a separate entity, ignoring all spatial interactions between pixels. Consequently, we used the SSIM as the loss between $I^{c}$ and $G_{2}\left(I^{g}\right)$. It was implemented and tested using perceptual quality metrics related to the visual perception of the human brain. The ratings of human subjects were used for validation. The SSIM assesses picture quality from the perspective of human visual perception, making it more suitable for loss function. The SSIM index was derived using common-size windows, x and y, between the pictures. Combining (2)–(4), we optimized the total loss for the generator as follows:

(5) $$L_{G}=\lambda_{l1}l_{1}+\lambda_{per}l_{VGG/i.j}^{Rec}+\lambda_{PIQE}l_{PIQE}+\lambda_{SSIM}l_{SSIM}+L_{G^{Ra}},$$

where $L_{G^{Ra}}$ is the adversarial loss, which we discuss in Section 3.4, and $\lambda_{l_{1}}$, $\lambda_{per}$, and $\lambda_{SSIM}$ are the coefficients used to balance the various loss terms.

### 3.3. Generator Architecture

**Figure 3:**
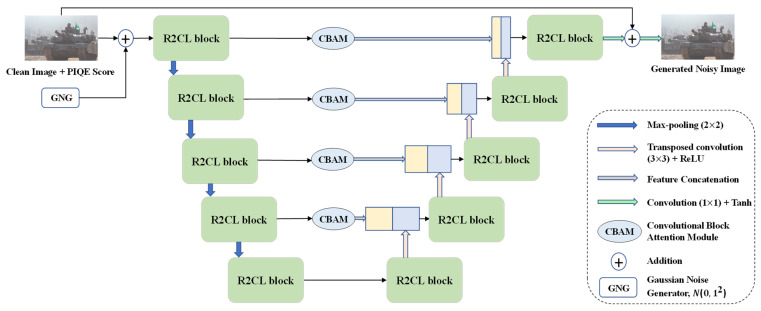
Generator architecture containing convolutional encoding and decoding units based on the recurrent residual convolutional layer (R2CL) and convolutional block attention module (CBAM) replacing the skip connection. GNG denotes the gaussian noise generator initially generating input random vector that is spatially repeated to the clean image.

Figure 3 shows the architecture of the generator in the proposed NG-GAN. Similar to GAN application [46], we sample random gaussian noise from $N\left(0,\;1^{2}\right)$, then add to pixel coordinates of the clean image to produce a random distribution that will result in the generation of various noisy photos of the same scenario. Two recurrent residual convolutional blocks were proposed within the recurrent residual convolutional layer (R2CL) of the proposed generator. In the encoding path, within each R2CL block, the features extracted from one convolutional layer are passed through a channel attention block, which contains a global average pooling layer followed by a 1-D channel attention layer, used to effectively capture channel correlation and prevent information loss. A recurrent convolutional block with a residual unit without a channel attention block was used in the decoding stages. Second, the CBAMs are used for adaptive feature refinement instead of skip connections. Finally, in the upsampling process, batch normalization (BN) was employed to improve the stability of the network and accelerate convergence [47]. Every stage in the encoding process includes a recurrent residual convolutional unit, which is composed of two 3 × 3 convolutions and incorporates recurrent connections to every convolutional layer to improve the model capacity to integrate contextual data. In addition, to construct more efficient and deeper models, residual connections were introduced. The set of feature maps was doubled, and the size was reduced by half each time a recursive residual convolutional unit was processed.

**Figure 4:**
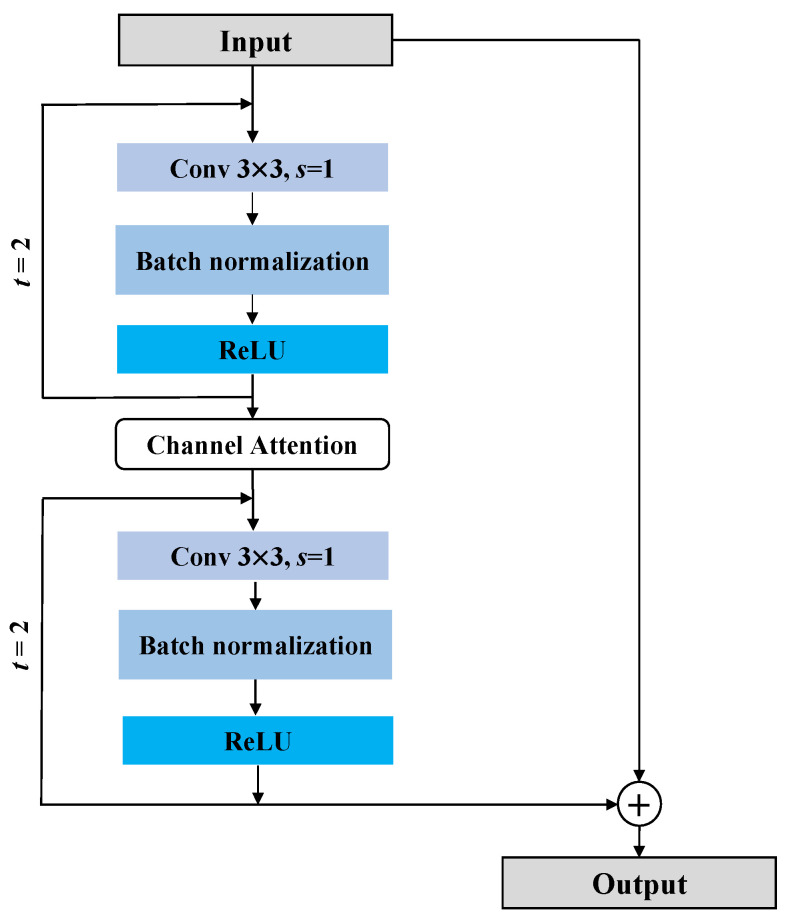
Architecture of the R2CL blocks in the NG-GAN generator. It has two blocks each consisting of 3 × 3 convolutional layers followed by a batch normalization and a ReLU activation layer in each block. The value t=2 indicates that the recurrent convolution layers are expanded to two-time steps.

Figure 4 shows the architecture of the R2CL block. In the R2CL, recurrent convolutional layers are applied in discrete time steps, as specified by the recurrent convolutional neural network (RCNN). Consider $p_{l}$ as an input sample at the $l^{th}$ layer of a block in the R2CL and $\left(i,j\right)$ as a pixel in an input sample of the $k^{th}$ feature map in the recurrent convolutional layer (RCL). Then, output $X_{ijk}^{l}\left(t\right)$ at time step t is given by (6):

(6) $$X_{ijk}^{l}\left(t\right)={\left(w_{k}^{f}\right)}^{T}p_{l}^{f\left(i,j\right)}\left(t\right)+{\left(w_{k}^{r}\right)}^{T}p_{l}^{r\left(i,j\right)}\left(t-1\right)+b_{k},$$

where $p_{l}^{f\left(i,j\right)}\left(t\right)\;$ and $p_{l}^{r\left(i,j\right)}$ are the standard convolutional layers and input sample to the $l^{th}$ RCL, respectively. The RCL generated from the $k^{th}$ feature map and standard convolutional layer are weighted by $w_{k}^{r}$ and $w_{k}^{f}$, respectively, where $b_{k}$ denotes bias. The standard ReLU function *f* () activates the output of the RCL, expressed as in (7).

(7) $$F\left(p_{l},w_{l}\right)=f\left(X_{ijk}^{l}\left(t\right)\right)=max\;\left(0,X_{ijk}^{l}\left(t\right)\right)$$

The output generated by the R2CL unit is given by (8).

(8) $$p_{l+1}=p_{l}+F\left(p_{l},w_{l}\right),$$

where the input of the R2CL layer is denoted by $p_{l}$ and $p_{l+1}$, which represent both the results derived from the downsampling and upsampling layers from the encoding and decoding paths, respectively. $F\left(p_{l},w_{l}\right)$ is the output from the $l^{th}$ layer of the RCNN.

**Figure 5:**
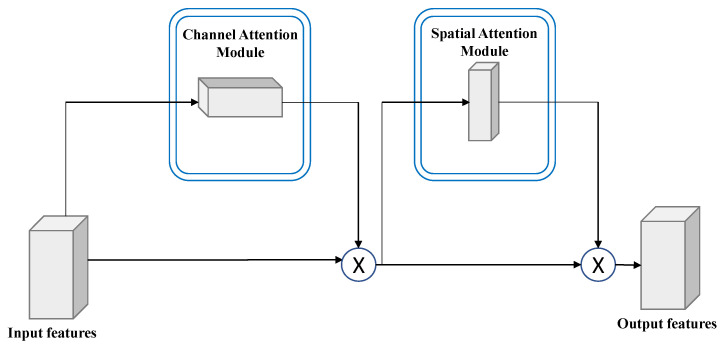
Convolutional block attention module (CBAM) block. It consists of channel and spatial attention modules. The feature maps from the encoding layers are refined through the CBAM block.

The upsampling operation related to the output derived from the R2CL unit was performed for each phase of the decoding path. After applying the upsampling technique, the feature maps are reduced by 50 percent, and the size is increased twice. The feature map size is reconstructed to the actual input image size in the final layer of the decoding path. As shown in Figure 3, the result from the BN layer is fed to the CBAM [35]. The CBAM consists of two sequential modules: the channel and spatial modules. The outputs generated from max-pooling and average pooling are combined and used by the channel submodule, whereas the spatial submodule adapts the same two outputs, which are pooled in terms of the channel axis and fed to the convolution layer. The intermediary feature map is refined using the CBAM module in each block of the deep network. The refined feature map is then concatenated with the feature maps obtained from the transpose convolution operation (Figure 5).

### 3.4. Discriminator Architecture

In our proposed architecture, we improve the discriminator using a relativistic GAN [31], which differs from the standard discriminator D. This was used to improve the discriminator performance. A relativistic discriminator aims to estimate the likelihood that a real image is more realistic than a fake one better than the conventional discriminator *D*, which estimates the likelihood that an input image is real. The relativistic discriminator aims to estimate the likelihood that real image $i_{r}$ is more realistic than generated image $i_{f}$.

The standard discriminator is expressed as $D\left(x\right)=\sigma \left(C\left(x\right)\right),$ where $\;C\left(x\right)$ is the non-transformed output from the discriminator and σ is the sigmoid function. The relativistic average discriminator $D_{Ra}$ is expressed by (9):

(9) $$D_{Ra}\left(i_{r},i_{f}\right)=\sigma \left(C\left(i_{r}\right)-E_{i_{f}}\left[C\left(i_{f}\right)\right]\right),$$

where $i_{r}$ is the real noisy image, $i_{f}$ is the fake noisy image, and $E_{i_{f}}$ is an average operator on all generated data in a minibatch. The discriminator loss is defined by (10):

(10) $$L_{D^{Ra}}=-E_{i_{r}}\left[\log\left(D_{Ra}\left(i_{r},i_{f}\right)\right)\right]-E_{i_{f}}\left[\log\left(1-D_{Ra}\left(i_{f},i_{r}\right)\right)\right],$$

and the generator adversarial loss is defined by (11):

(11) $$L_{G^{Ra}}=-E_{i_{r}}\left[\log\left(1-D_{Ra}\left(i_{r},i_{f}\right)\right)\right]-E_{i_{f}}\left[\log\left(D_{Ra}\left(i_{f},i_{r}\right)\right)\right]$$

The adversarial loss of the generator includes both $i_{r}$ and $i_{f}$. Consequently, in adversarial training, the generator updates itself according to the discriminators’ output of both fake and actual data. 

## 4. Experimental Results

We set the values of the coefficients $\lambda_{l_{1}}$ = 5.0, $\lambda_{per}r$ = 0.08,$\;\lambda_{PIQE}$ = 0.3, and $\lambda_{SSIM}$ *=* 0.1, which were empirically determined based on many experimental trials. All submodules were trained with the Adam optimizer, with $\beta_{1}$ = 0.5 and $\beta_{2}$ = 0.999. The images were cropped to a size of 64 × 64 pixels and fed to the model. The batch size was set to 1. We cropped 17,000 patches with a size of 64 × 64 pixels from clean and noisy images and sampled those images to train the model; horizontal and vertical flips and random rotations 90 × θ, where θ = 0, 1, 2, 3, were performed for data augmentation. We added patches extracted from old noisy images to the clean images to collect more noisy images and extracted the patches using a noise block extraction algorithm [20]. During the training phase, the learning rate was set to 1 $\times 10^{-5}$. After every 14 epochs, the learning rate was reduced by multiplying with 0.8 for model stabilization. All models were trained on a GeForce RTX3090 GPU.

### 4.1. Datasets

To train the proposed model, we use high-quality clean images from REDS [38], PASCAL VOC [39], and DIV2K [40] datasets. REDS contains 240 videos, each video with 100 frames, so it contains a total of 24,000 clean images. The PASCAL VOC dataset contains 17,125 high-quality clean images, and DIV2K contains 800 high-quality clean images. We collected noisy images by extracting frames from old movies from the 1920s–1970s as noisy samples, and we also distorted clean images by adding Gaussian blur, JPEG compression, and adding the noisy patches that were extracted from old videos using the noise estimation method [20]. 

**Figure 6:**
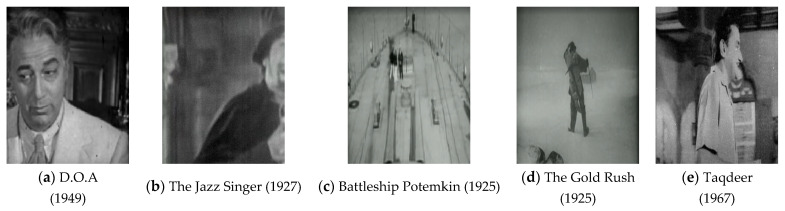
Examples of old noisy video frames collected from old movies from the 1920s–1970s, which illustrates the presence of artifacts and noise pattern in old video frames.

Figure 6 shows five old noisy images collected from movies from the 1920s–1970s. The old images in the film are contaminated with complicated degradation noise, which is different from synthetic noise and difficult to model mathematically. The noise types in the old movies include compression artifacts from compression algorithms, blur noise that occurs due to improper camera lens alignment, unstructured defects such as film grain, color fading, and structured defects, e.g., scratches and dust spots. Hence, replicating these noisy patterns is more difficult compared to the digital noise in modern images.

### 4.2. Qualitative Comparison of Denoised Videos

The datasets generated by C2N [45], CycleGAN [29], and the proposed NG-GAN were validated using SOTA denoising networks: BasicVSR [40] and BasicVSR++ [48]. These two SOTA denoisers exhibit the best performances in image denoising. The effectiveness of the architectures was validated through a qualitative comparison of the PSNR and SSIM values. For comparison, C2N, CycleGAN, and NG-GAN were trained under the same datasets and conditions, and the same number of paired datasets from each generating architecture was obtained. Finally, BasicVSR and BasicVSR++ were trained using the generated datasets, and the old videos were tested on the BasicVSR and BasicVSR++ trained by C2N, CycleGAN, and NG-GAN, as well as the pretrained BasicVSR. 

**Figure 7:**
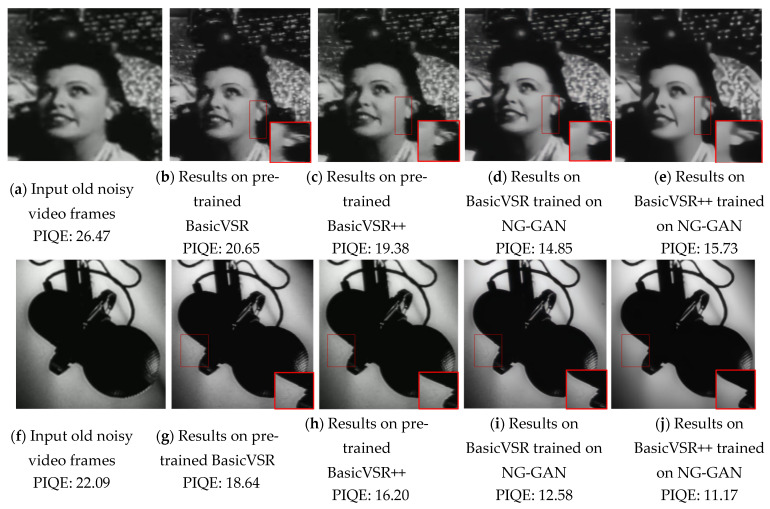
Examples of de-oldifying old videos using pretrained SOTA methods and SOTA methods trained on NG-GAN generated dataset. (**a**) Old video frames, (**b**) de-oldified output from pre-trained BasicVSR, (**c**) de-oldified output from pre-trained BasicVSR++, (**d**) de-oldified output from BasicVSR trained on the proposed NG-GAN generated datasets, and (**e**) de-oldified output from BasicVSR++ trained on the proposed NG-GAN-generated datasets.

Figure 7 shows a visual comparison of the results between the denoisers trained on the datasets generated by the proposed model and the pre-trained denoisers (BasicVSR and BasicVSR++). The pre-trained BasicVSR and BasicVSR++ models were trained on REDS [38] and Vimeo-90K [49] datasets, which contain images distorted by blur, JPEG compression artifact, digital camera noise, etc. with high-quality clean and their paired noisy images. Figure 7a,f are input images, and Figure 7b,g are generated noisy images. Figure 7 shows that the video restorers trained on our model-generated datasets can produce significantly better-denoised images than those trained on REDS, which include synthetic noise with a Gaussian distribution. As shown in Figure 7, BasicVSR and BasicVSR++ trained on datasets generated by the proposed NG-GAN can preserve the texture, details, and edges of the images, whereas the pretrained models show lower-quality results, as shown in Figure 7b,c. This is because the pretrained models were trained using synthetic Gaussian and Poisson noise models, which do not reflect the actual old image noise and artifact patterns. Thus, they fail to capture the noise distribution of the old videos well. The marked region in Figure 6 highlights the restored region from the pretrained BasicVSR and BasicVSR++ and BasicVSR and BasicVSR++ trained on the datasets generated by the NG-GAN. The highlighted region in the first row clearly shows the delineation of the ear and neck region, maintaining edges and other structures intact. Notably, the restorers trained on our dataset generated by the NG-GAN can achieve smooth and highly denoised images compared with those pre-trained (Figure 7). The PIQE values of old noisy frames, video frames denoised by pre-trained BasicVSR and BasicVSR++, and video frames denoised by BasicVSR and BasicVSR++ trained on the datasets by the proposed NG-GAN were calculated, respectively. It is observed that the denoisers trained on the datasets which are generated by the proposed NG-GAN can show better denoising capability with lower PIQE values compared to the pre-trained denoisers

### 4.3. Quantitative Comparisons for Denoised Old Images

**Figure 8:**
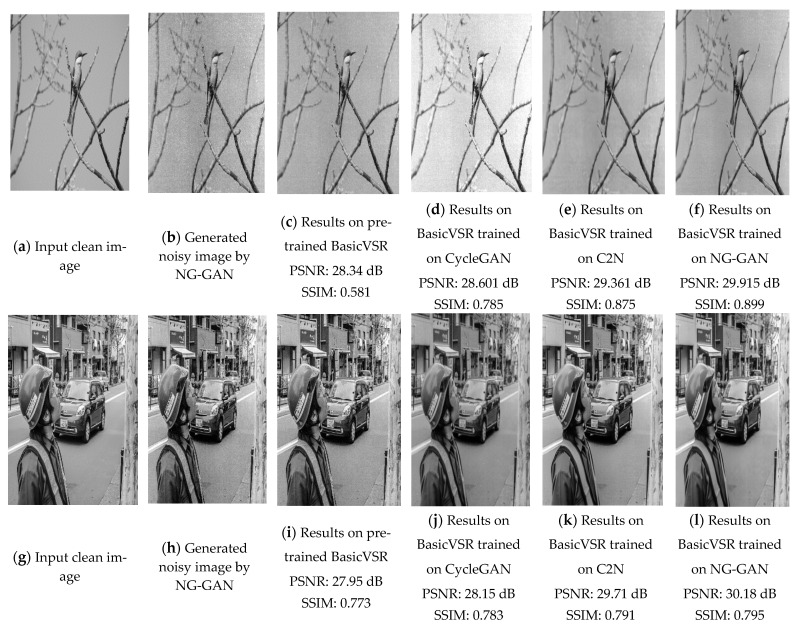

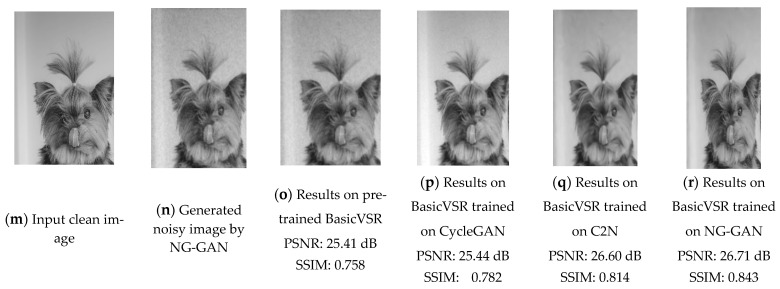
Examples of oldifying and de-oldifying videos using pre-trained SOTA methods, SOTA methods trained on C2N and NG-GAN generated dataset. (**a**,**g**,**m**) High quality images form Flickr 2 k image dataset. (**b**,**h**,**n**) Oldified noisy frames generated by NG-GAN. (**c**,**i**,**o**) De-oldification output from pre-trained BasicVSR. (**d**,**j**,**p**) De-oldification output from BasicVSR++ trained on CycleGAN generated dataset. (**e**,**k**,**q**) De-oldification output from BasicVSR++ trained on C2N generated dataset. (**f**,**l**,**r**) De-oldification output from BasicVSR++ trained on NG-GAN generated dataset.

In Figure 8, experiments were performed to test the results of the denoiser, trained using various datasets, including REDS, the C2N-generated, the CycleGAN-generated, and the proposed NG-GAN-generated datasets. The metrics used to measure the quality of the datasets are the PSNR and SSIM values. The images in Figure 8b,h,n show noisy images produced by the proposed NG-GAN model. The results in Figure 8c,i,o show the images denoised by the pretrained BasicVSR model. Then, these outputs denoised by the pretrained BasicVSR model were compared with the outputs denoised by the BasicVSR trained on the datasets generated by the NG-GAN, in terms of the PSNR and SSIM metrics. As shown in the third and the sixth columns, the outputs trained using our NG-GAN-generated datasets show outperforming results in PSNR and SSIM values. Likewise, the outputs trained using the datasets using C2N-generated and CycleGAN-generated datasets show lower PSNR and SSIM values. In addition, the output images trained on the NG-GAN datasets show subjectively better results, as shown in Figure 8f,l,r. This proves the effectiveness of the NG-GAN-generated dataset.

We evaluated the average performance of the SOTA denoising methods, BasicVSR, BasicVSR++, GCBD, and UIDNet on datasets generated by the proposed NG-GAN, C2N, CycleGAN, BasicVSR and BasicVSR++ are known as the best-performing denoisers among the supervised denoising architectures, and GCBD and UIDNet are the unsupervised denoisers to show the best results. This experiment is to investigate how the generated datasets can train the denoiser well. Table 1 shows PSNR and SSIM values on average for each denoising method when they are trained using various datasets. As shown in Table 1, BasicVSR and BasicVSR++ trained using the NG-GAN generated datasets achieve significantly better PSNR and SSIM values. 

**Figure 9:**
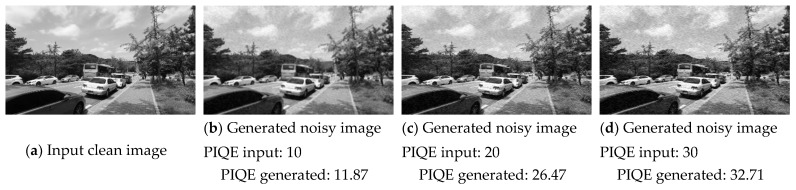

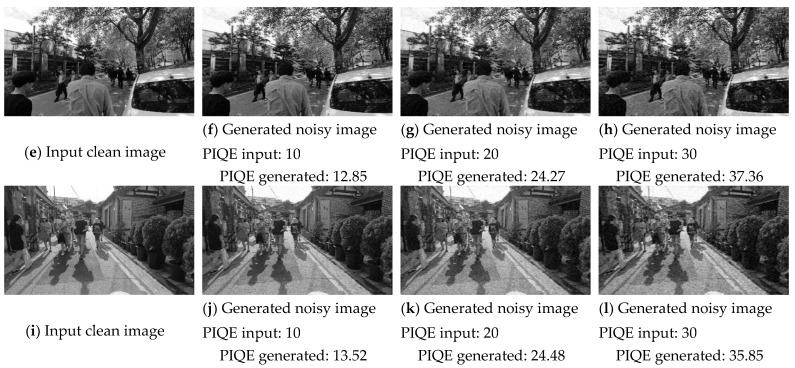
Effectiveness of various PIQE values as input for the generator. (**a**,**e**,**f**) are the clean images from REDS dataset. (**b**–**d**), (**f**–**h**), and (**j**–**l**) are the images generated by the proposed model with different PIQE values as an input.

We investigated the impact of the PIQE value on the REDS dataset [33] by testing various PIQE values. Figure 9 shows that, with a value of 10, the image shows a controlled amount of noise and distortion in Figure 9b,f,j. With an increase in the PIQE value, the distortion and noise increased in proportion to the input PIQE value, as shown in Figure 9c,d,g,h,k,l. This is because the NG-GAN was trained using the PIQE value extracted from the old, degraded images. This helps the model to learn noise generation better. The PIQE values are provided to the generator as input with the clean images, resulting in the PIQE values of the generated distorted image.

**Figure 10:**
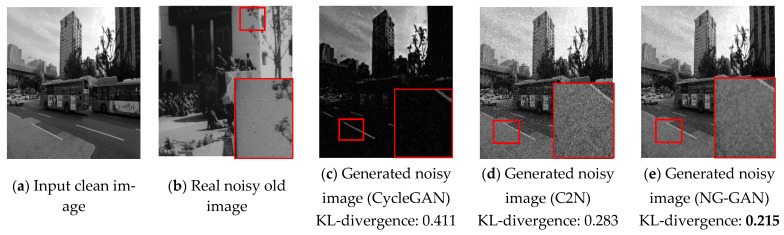

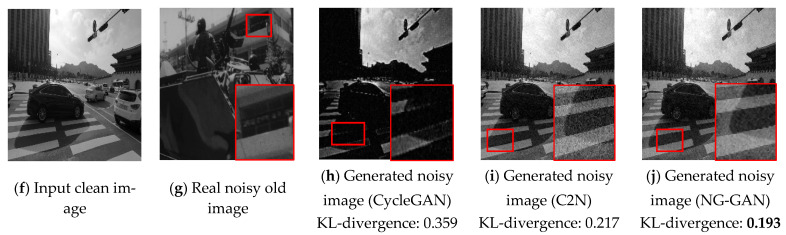
Visual comparison of noisy images generated by CycleGAN, C2N, and NG-GAN. (**a**) and (**f**) are high-quality images from the REDS dataset, (**b**,**g**) are noisy images generated by CycleGAN, (**c**,**h**) are noisy images generated by C2N, (**d**,**i**) are noisy images generated by the NG-GAN, and (**e**,**j**) are real noisy images from old videos.

Figure 10 shows examples of noisy images generated by CycleGAN, C2N, and the proposed NG-GAN method, respectively. The images in Figure 10a,f are clean input images, Figure 10b,g are actual old noisy images, and the images in Figure 10c–e,h–j are the images generated by CycleGAN, C2N, and NG-GAN, respectively. As can be seen in Figure 10, the proposed NG-GAN can generate more realistic-looking old image noise, whereas other noise generation networks fail to generate old image noise with actual noisy patterns in the given clean images. The image generated by CycleGAN shows unclear output, and the edges of the objects are not retained well, as shown in the red-marked region in Figure 10. We calculated the KL-divergence [50] between the real noisy image and generated noisy image by extracting a smooth region from the real noisy image and the generated noisy image. In general, KL-divergence measures the difference between two probability distributions. Hence, the lower value of KL-divergence indicates a higher similarity between the two populations of images. Statistically, the proposed NG-GAN achieves the lowest KL-divergence values between the generated noisy image and the real noisy image compared to other noisy image generators.

Table 2 shows the KL-divergence values calculated between the noise map of old images and the noise map of images generated by CycleGAN, C2N, and our proposed method. As shown in Table 2, it is observed that our proposed method achieves the lowest KL-divergence between the actual old noisy images and generated noisy images. The lower KL-divergence indicates that the proposed model is successfully generating the old image noise pattern.

### 4.4. Ablation Study

In order to investigate the efficiency of particular parts of the proposed architecture, we performed an ablation study. Table 3 shows the lists of ablation studies for network components and loss functions in our proposed method. For comparison, we set the CycleGAN architecture as the baseline method. In method (a), we incorporated the proposed R2CL generator architecture and used two types of loss with VGG-19 and SSIM losses to see if our model can show better performance compared with the baseline method in terms of the PIQE metric. It can be observed that the PIQE metric increases to 24.49, which indicates that the model can generate better-quality noise than the baseline model. This is because the inclusion of the R2CL generator can effectively capture channel correlation and prevent information loss. Additionally, it can successfully imitate the noisy pattern of degraded images. Furthermore, the VGG-19 and SSIM losses can guide the network well in generating old noisy images while maintaining the visual quality and structure of the images, which in turn helps to increase the PIQE value. In method (b), we tested CBAM to see any change in the PIQE metric compared with method (a). It is observed that incorporating CBAM can increase the PIQE value because of its ability to focus on the most informative aspects while creating degraded images. Moreover, this helps to retain image features and edges while generating old image noise, which creates the realistic noisy image. Finally, in method (c), we included the PIQE metric and PIQE loss in addition to the method (b) to show the effectiveness of the PIQE value. We observed that method (c) yields the highest PIQE value compared to other methods. Since the PIQE value indicates the amount of noise and distortion in an image, extracting the PIQE value from the old image and concatenating it with the clean image provides the network with additional information about the amount of noise generation. Additionally, PIQE loss is considered to evaluate the distortion in the generated noisy image and the actual old noisy image. Thus, this time, the model can effectively generate realistic noisy patterns without the use of any paired dataset.

## 5. Conclusions

This paper proposes a model that can effectively produce old noisy images by imitating the noise distribution of old images. Since it is difficult to obtain a number of paired datasets of old images, denoising such images using the supervised deep learning models is very challenging. Thus, most existing studies have not considered solving this problem. To solve this issue, we proposed a novel framework, NG-GAN, that replicates the noise distribution of deteriorated old images using unpaired datasets and a no-reference PIQE metric, which can guide the network in generating noisy images. A recurrent residual convolutional and attention mechanism-based generator is proposed in the NG-GAN framework to successfully generate the noisy pattern of degraded images. Using the dataset generated by the NG-GAN, video restorers can better learn to denoise old, degraded images. We show that the state-of-the-art denoiser can achieve higher PSNR and SSIM values when datasets generated by our proposed model are used as training datasets, compared to ones generated by other noise generation methods. Our approach can successfully imitate crucial degraded noise patterns of actual old images from the given clean images. 

## Figures and Tables

**Table 1:** Comparison of old noisy images denoised by state-of-the-art denoisers and image restorers trained on the dataset generated by our proposed model.

Models		PSNR (dB)	SSIM
BasicVSR	Pretrained BasicVSR [32]	24.91	0.703
	BasicVSR (CycleGAN) [18]	24.93	0.698
	BasicVSR (C2N) [45]	25.27	0.736
	BasicVSR (Proposed NG-GAN)	**25.48**	**0.739**
BasicVSR++	Pretrained BasicVSR++ [50]	25.21	0.727
	BasicVSR++ (CycleGAN) [18]	25.03	0.705
	BasicVSR++ (C2N) [45]	25.81	0.768
	BasicVSR++ (Proposed NG- GAN)	**25.89**	**0.781**
Others	GCBD [44]	24.22	0.726
	UIDNet [14]	25.17	0.694

**Table 2:** Average values of Kullback–Leibler (KL) divergence between generated and real noisy images.

Metric	CycleGAN	C2N	NG-GAN (Proposed Method)
KL-divergence	0.3436	0.2195	**0.1879**

**Table 3:** Results for ablation study. (a) Baseline (CycleGAN Network), (b) only R2CL generator in the proposed NG-GAN model, (c) R2CL generator with CBAM in the proposed NG-GAN model, (d) R2CL generator with CBAM and PIQE metric calculated from old images concatenated to the clean images in the proposed NG-GAN model.

Methods	Proposed NG-GAN								PIQE
	Network			Loss Functions					
	R2CL	CBAM	PIQE Guided	Cycle Consistency Loss	VGG-19 Loss	PIQE Loss	SSIM Loss	Discriminator Loss	
Baseline (CycleGAN)	✖	✖	✖	✓	✖	✖	✖	✓	22.73
(a)	✓	✖	✖	✓	✓	✖	✓	✓	24.49
(b)	✓	✓	✖	✓	✓	✖	✓	✓	27.36
(c)	✓	✓	✓	✓	✓	✓	✓	✓	29.18

## Data Availability

Not applicable.
